# The cost effectiveness of a multidomain intervention on physical, cognitive, vascular, dietary and psychosocial outcomes among community dwelling older adults with cognitive frailty in Malaysia: The AGELESS Trial

**DOI:** 10.1002/alz70860_106477

**Published:** 2025-12-23

**Authors:** Pavapriya Ponvel, Suzana Shahar, Devinder Kaur Ajit Singh, Arimi Fitri Mat Ludin, Ponnusamy Subramaniam, Norhayati Ibrahim, Haron Hasnah, Aniza Ismail, Chin Ai‐Vyrn, Mazlyfarina Mohamad, NURUL HIDAYAH MD FADZIL, Norhayati Mustafa Khalid, Aisyah Mohammad Safien, Jamilah Mohammad Hanipah, Azyana Ibrahim, Jenni Lehtisalo, Miia Kivipelto, Francesca Mangialasche

**Affiliations:** ^1^ Center of Wellness and Healthy Aging, Faculty of Health Sciences, Universiti Kebangsaan, Kuala Lumpur, NA, Malaysia; ^2^ Centre for Healthy Aging and Wellness, Faculty of Health Sciences, Universiti Kebangsaan Malaysia, Kuala Lumpur, Malaysia; ^3^ Programme of Physiotherapy & Centre for Healthy Ageing and Wellness (HCARE), Faculty of Health Sciences, Universiti Kebangsaan Malaysia, Kuala Lumpur, Malaysia; ^4^ Programme of Biomedical Science & Centre for Healthy Ageing and Wellness (HCARE), Faculty of Health Sciences, Universiti Kebangsaan Malaysia, Kuala Lumpur, Malaysia; ^5^ Clinical Psychology Programme and Centre for Healthy Aging and Wellness, Faculty of Health Sciences, Universiti Kebangsaan Malaysia, Kuala Lumpur, Kuala Lumpur, Malaysia; ^6^ Center of Wellness and Healthy Aging, Faculty of Health Sciences, Universiti Kebangsaan, Kuala Lumpur, Malaysia; ^7^ Department of Public Health Medicine Faculty of Medicine, Universiti Kebangsaan, Kuala Lumpur, Malaysia; ^8^ Department of Medicine, Faculty of Medicine, Universiti Malaya, Kuala Lumpur, Malaysia; ^9^ Centre for Diagnostic, Therapeutic & Investigative Studies, Faculty of Health Sciences, Universiti Kebangsaan, Kuala Lumpur, Malaysia; ^10^ Centre for Healthy Ageing and Wellness (H‐Care), Faculty of Health Science, National University of Malaysia, Kuala Lumpur, Malaysia; ^11^ Institute of Public Health and Clinical Nutrition, University of Eastern Finland, Kuopio, Finland; ^12^ Finnish Institute for Health and Welfare, Helsinki, Finland; ^13^ Karolinska University Hospital, Theme Inflammation and Aging, Stockholm, Sweden; ^14^ Department of Neurobiology, Care Sciences and Society, Karolinska Institutet, Stockholm, Sweden; ^15^ Institute of Public Health and Clinical Nutrition, University of Eastern Finland, Kuopio, Kuopio, Finland; ^16^ Imperial College London, London, United Kingdom; ^17^ FINGERS Brain Health Institute, Stockholm, Sweden; ^18^ Division of Clinical Geriatrics, Center for Alzheimer Research, Department of Neurobiology, Care Sciences and Society, Karolinska Institutet, Stockholm, Sweden; ^19^ Karolinska University Hospital, Theme Inflammation and Aging, Solna, Sweden

## Abstract

**Background:**

Cognitive frailty (CF) in older adults is linked to increased dementia risk, but is a potentially reversible syndrome that may benefit from lifestyle‐based multidomain interventions. The AGELESS randomized controlled trial (RCT) assessed the effect of a 2‐year multidomain intervention on cognitive, physical, vascular, dietary, and psychosocial outcomes, and its cost‐effectiveness, in a Low‐Middle‐Income Country (LMIC), Malaysia.

**Method:**

106 community‐dwellers from Klang‐Valley, Malaysia, aged 60+ years and meeting criteria for (pre)‐CF (≥ 1 Fried's criteria and Clinical Dementia Rating scale=0.5) were randomized 1:1 to a culturally adapted multidomain intervention ‐ physical activity, cognitive training, nutritional and psychological counselling, cardiovascular care – or to an educational module. Primary outcomes, assessed at baseline, 12 and 24 months, included the modified Neuropsychological Tests Battery (mNTB) and physical performance. Intervention costs were calculated to determine Incremental Cost‐Effectiveness Ratios (ICERs). An intention‐to‐treat analysis was conducted.

**Result:**

The trial occurred during the COVID‐19 pandemic, with a 50% drop‐out rate over 2‐years. Yet, adherence among participants was over 50% for all intervention components (range 53%‐91%). Significant intervention effects were observed in mNTB primary outcomes including verbal memory (verbal paired associates), visual memory (visual paired associates), attention and working memory (digit span test), lower body flexibility (chair sit and reach), walking speed (6 meter walk test), cardiovascular endurance (2‐minute step test) (for all measures *p* <0.05 for interaction time*group in repeat‐measures ANOVA). Changes in (pre)‐CF status were not significantly different between intervention and control. Total cost of the AGELESS intervention for 2‐years was 356USD per subject (intervention arm) and 109USD in the control arm. The ICER computation showed that 2‐minute step test was the most cost effective (34USD), followed by chair sit and reach (63USD) and verbal paired associates delayed recall (113USD).

**Conclusion:**

Multidomain lifestyle‐based interventions can be cost‐effective, sustainable preventive approaches to promote healthy aging and reduce dementia risk. AGELESS is part of the Worl‐Wide‐FINGERS global network of multidomain interventions for dementia risk reduction, and offers a successful model to develop preventive interventions in LMICs, where there is a larger prevention potential due to prevalent lifestyle‐related risk factors.

